# Spanish adaptation and psychometric properties of the child version of the Cognitive Emotion Regulation Questionnaire

**DOI:** 10.1371/journal.pone.0201656

**Published:** 2018-08-02

**Authors:** Mireia Orgilés, Alexandra Morales, Iván Fernández-Martínez, Juan Manuel Ortigosa-Quiles, José P. Espada

**Affiliations:** 1 Department of Health Psychology, Miguel Hernández University, Elche, Spain; 2 Personality, Evaluation and Psychological Treatment, University of Murcia, Murcia, Spain; Hong Kong Polytechnic University, HONG KONG

## Abstract

**Background:**

The Cognitive Emotion Regulation Questionnaire for children (CERQ-k) is a useful clinical and research tool to identify cognitive patterns of emotion regulation that predict the presence of emotional symptomatology. This study aimed to validate the Spanish version of the CERQ-k (the CERQ-Sk) using a sample of children from Spain, which is not available.

**Methods:**

The sample consisted of 582 children (48.6% girls) aged between 7 and 12 years (*M*_age_ = 9.49; *SD* = 1.2) recruited from Alicante, Spain. Cognitive emotion regulation strategies, anxiety and depressive symptomatology were self-reported evaluated. Factor structure, internal consistency, temporal stability with the Spanish version for children were examined. Convergent validity was evaluated using Spearman correlations to examine the relationships between the CERQ-k and measures of anxiety (trait anxiety subscale of STAI-C) and depression (CDI).

**Results:**

The Spanish version of CERQ-Sk had the same nine factors proposed in the original version. Ordinal alpha of the total scale was excellent (.88), and moderate indexes were found for each subscale (.56 to .75). The 8-week test-retest coefficient was adequate for the total scale (ICC = .74) and moderate for the subscales (.54 to .70). Evidence of convergent validity was provided through correlations with the CDI (depression) and trait anxiety subscale of the STAI-C (anxiety). Cognitive strategies such as Rumination, self-blame, catastrophizing, and other-blame were significantly and positively related to depressive and anxiety symptoms. Moreover, positive refocusing and planning seemed to act as strategies that have a positive effect on the prevention of depression in children.

**Conclusions:**

Results suggest that the CERQ-Sk is a reliable and valid tool that can be useful for researchers and clinicians to identify maladaptive cognitive emotion regulation patterns that may increase the risk of emotional problems, and orient treatment and prevention of mental health problems in children from Spanish-speaking countries.

## Introduction

Emotion regulation is an essential process in the daily life of human beings that allows us to use different strategies to modify the course, intensity, duration and expression of emotional experiences depending on the situation or our goals [1]. Research on cognitive strategies in emotion regulation, regardless of behavioural factors and the influence of these on the presence of psychopathology, has become of great importance. These strategies can be defined as conscious cognitive processes aimed at controlling and regulating emotions in threatening situations [2].

As children grow older, their repertoire of emotion regulation strategies gradually increases, from the use of primarily behavioural strategies to cognitive strategies [3]. There is evidence that childhood is an important stage in which children's emotional control increases, reducing negative emotions over the years [4]. However, the type of cognitive emotional regulation (CER) strategies developed in infancy will have repercussions for the style of cognitive coping used for stressful situations in adolescent and adult life, where CER strategies have been widely related with the presence of psychopathology [5]. As in adults, the use of certain CER strategies are strongly related to the presence of emotional problems in children (i.e., anxiety and depression), having these strategies a potential value as an indicator of child psychopathology and as a target for the prevention and treatment of these problems [6, 7]. In this regard, it has been stressed that the development of adaptive CER strategies during childhood can have an important protective effect and reduce the risk of future disorders [8]. Therefore, the assessment of CER strategies can be important as it can be useful for the diagnosis process of childhood emotional problems and for establishing effective prevention or intervention strategies [8].

There are several instruments that evaluate emotion regulation processes, such as the Difficulties in Emotion Regulation Scale (DERS) [9], Emotion Regulation Questionnaire (ERQ) [10], Trait Meta-Mood Scale (TMMS) [11] and the Negative Mood Regulation Scale (NMR) [12]. However, the Cognitive Emotion Regulation Questionnaire (CERQ) [13] is the only questionnaire that focuses on evaluating purely cognitive strategies of emotion regulation, without encompassing the broad repertoire of intrinsic and extrinsic strategies for control, evaluation and modification of emotions.

The CERQ was initially developed by Garnefski et al. [13] as a self-report tool to evaluate the cognitive strategies of emotion regulation that the person uses in response to threatening or stressful situations. This multidimensional questionnaire consists of 36 items that are answered on a 5-point Likert scale, in which 1 corresponds to "almost never" and 5 to "almost always." The nine scales that comprise this instrument have 4 items each and correspond to the following cognitive strategies: a) *Self-blame*: thoughts that attribute the cause of the negative event and the emotion to oneself; b) *Acceptance*: to resign and accept the irreversibility of the negative experience; c) *Rumination*: state of excessive worry by negative thoughts and feelings; d) *Positive focusing*: having pleasant and joyous thoughts different from the negative event; e) *Planning*: thinking about how to solve the problem; f) *Positive reappraisal*: thoughts that highlight some positive aspect of the unpleasant event; g) *Putting into perspective*: decrease and relativize the severity of the event; h) *Catastrophizing*: to think about the horrible thing of what happened and conclude that it is the worse experience lived, even compared with what other people have experienced; and i) *Other-blame*: make others responsible for the negative event that happened.

In the Spanish adaptation of the CERQ, Domínguez-Sánchez, Lasa-Aristu, Amor, and Holgado-Tello [14] proposed an alternative model in which these nine cognitive strategies constitute the first order factors and can be grouped into two second order factors: adaptive strategies (putting into perspective, acceptance, positive reappraisal, positive refocusing, and planning) and maladaptive strategies (rumination, catastrophizing, self-blame, and other-blame). From this classification, also raised by the authors of the original CERQ [13], numerous studies have found that the use of maladaptive strategies are strongly negatively related to the symptoms of anxiety and depression, both in adult and adolescent populations [15–17] and in children [6, 7, 18]. In this regard, a recent review found that adaptive emotional regulation strategies were negatively associated with symptoms of anxiety and depression in youth, while maladaptive strategies were positively associated, showing that the presence of more symptoms was associated with the use of maladaptive strategies and fewer symptoms with the use of adaptive strategies [5].

The original version of the CERQ was applied to a sample of adolescents and adults from the Netherlands [13, 19]. Subsequently, the CERQ has been translated and adapted in different countries such as France [20], China [21], Romania [22], Iran [23], Turkey [24], Spain [14], Argentina [25] and Peru [26], among others. However, these adaptations focused on the adult population. Both the original version of the CERQ and the reported adaptations have demonstrated good psychometric properties, as the authors identified acceptable levels of internal consistency reliability for each dimension, and evidence of criterion validity consistent with measurements of depression, anxiety, and positive and negative emotions; and all are consistent with the nine-factor structure.

Several studies with samples of adolescents have shown good psychometric properties: maintaining the original factor structure, internal consistency reliability coefficients ranging from .68 to .83, and a test-retest stability with Pearson *r* values between .40 and .60 [2, 13, 19]. Although the CERQ was first developed with adult and adolescent populations, a version adapted for children under 12 was created, the CERQ-k [6]. This version and its adaptations to other languages maintain the original factor structure and have shown adequate internal consistency, with Cronbach's alphas between .62 and .79 [6, 8]. Despite being an instrument of great clinical and research utility to detect cognitive patterns of emotion regulation that predict the presence of emotional symptomatology in children, the measure has not been validated for Spanish-speaking children. As such, the present research aimed to provide a new tool for the evaluation of cognitive strategies of emotion regulation for use with Spanish-speaking children.

The main objective of the present study was to adapt and validate the CERQ-k in a community sample of children between 7‒12 years of age in Spain. To achieve this aim, the factor structure was analysed using confirmatory factor analysis (CFA), internal consistency and test-retest reliability of each dimension of the CERQ-k were examined, and convergent validity was evaluated. Based on previous studies [6–8], it was hypothesized that the psychometric properties of the Spanish version of the CERQ-k would be adequate, and the nine factors corresponding to the nine cognitive strategies of coping would be confirmed.

## Methods

### Participants

The sample consisted of 582 children (48.6% girls) aged between 7 and 12 years (*M*_age_ = 9.49; *SD* = 1.2). The age distribution was as follows: 2.7% (*n* = 15) were 7 years old; 19.6% (*n* = 114) were 8; 31.8% (*n* = 185) were 9; 23.4% (*n* = 136) were 10; 17.9% (*n* = 104) were 11; and 4.6% (*n* = 28) were 12 years old. Most children had been born in Spain (98.6%) and the others were born in other Central European, North Asian, American, and South American countries, but all of them were Spanish-speaking. Middle socio-economic status was predominantly. The mean number of siblings of the participants was 1.16 (*SD* = 0.76). The children were recruited from 11 public and private schools in urban areas in the southeast of Spain.

### Instruments

#### Cognitive Emotion Regulation Questionnaire (CERQ-k) [6]

The CERQ-k is a self-report questionnaire that contains 36 items on nine different subscales that assess what children think after experiencing negative life events. The CERQ-k subscales include Self-Blame, Catastrophizing, Rumination, and Other-Blame (maladaptive CER strategies), and Positive Reappraisal, Planning, Positive Refocusing, Acceptance, and Putting into Perspective (adaptive CER strategies). Each subscale consists of 4 items rated on a 5-point Likert-type scale (1 = “almost never,” 5 = “almost always”). The higher the score on each scale, the more pronounced use of the respective cognitive coping strategy. The original kids version of the CERQ has demonstrated good psychometric properties, and all subscales have shown high internal consistency reliability coefficients ranging from .62 to .79 [6, 8].

#### Child Depression Inventory (CDI) [27]

The CDI assesses depressive symptomatology in children and adolescents ages 7 to 17 years old. The self-report questionnaire is composed of 27 items that are grouped into two subscales: Dysphoria (17 items) and Negative Self-Esteem (10 items). Items are scored from 0 (“No symptoms”) to 2 (“Depressive symptoms”), and children select one of three statements that best describes them in the past two weeks. Higher scores indicate considerably more severe depression. Del Barrio and Carrasco [28] validated the Spanish version of the questionnaire, finding satisfactory psychometric properties with an internal consistency reliability coefficient of .79.

#### State-Trait Anxiety Inventory for Children (STAI-C) [29]

Of the two 20-item scales of the STAI-C that measure state and trait anxiety in children, the trait anxiety subscale was used for this study. The scale assesses anxiety as a persistent emotional state. Each item is scored on a 4-point intensity scale (0 = “Not at all” to 3 = “Very much”). This tool can be used with 9‒15 year old children or with younger children with above average reading and comprehension ability. Higher scores indicate higher levels of anxiety. The Spanish version was validated by Pons-Salvador, Frías, and Del Barrio [30], who found the scale to have high reliability (α = .85‒.89) and support for concurrent validity based on correlations with other scales of anxiety (.75).

### Procedure

The cultural and language adaptation of CERQ-k for Spanish children was conducted following the guidelines for adaptation of scales of Muñiz, Elosua and Hambleton [31]. All necessary permissions for use of the CERQ being validated, including permissions to publish the items of the scale from Prof. Garnefski–the developer of CERQ–were obtained. Two bilingual child psychology therapists translated the CERQ-k from English into the Spanish language. The initial Spanish version of CERQ-k (CERQ-Sk) was translated back into English by an English-native. Other experts contributed by proposing alternative wording when it was necessary. Appropriateness and accuracy of the items was ensured by discussing possible discrepancies among experts involved in the adaptation of the scale to Spain. The final version was initially tested in a small group of children aged 7–12 (*n* = 7) to check if the items were understood and were appropriate. No modifications were needed.

Ethical approval from the ethics board of the Miguel Hernández University (DPS.MO.02.14) and permission of the participating schools was obtained. Written parental consent was obtained for all participating children. Participants completed the final form of CERQ-Sk and other measures on anxiety and depression collectively in groups of approximately 10 children. A researcher remained in each classroom to read aloud information about the study and resolve any questions. Questionnaires were reviewed to avoid missing data. Participation was voluntary and anonymous. No incentives were provided.

### Statistical analysis

In the first step of the validation process of the CERQ for children in Spain, the original nine subscales were confirmed using confirmatory factor analysis (CFA). Reference values were taken from Hu and Bentler [32] indicating a good model when the comparative fit index (CFI) and the Tucker–Lewis index (TLI) were greater than .90, and the root mean square error of approximation (RMSEA) value was lower than .08. The normality assumption of the items with the Kolmogorov-Smirnov statistic was not met (*p* < .05). Because of robustness in cases of non-normality and ordinal data, diagonally weighted least squares (DWLS) method was selected [33, 34]. Internal consistency reliability was estimated using ordinal alpha [35, 36]. The latent structure and internal consistency of the CERQ were examined using Lavaan package for structural equation modelling version 0.5–12 (BETA) [37].

Descriptive statistics were used to characterize the sample in the present study. Based on the nine CERQ scales, we examined its psychometric properties. Intra-class correlation (ICC) was used to explore test-retest reliability [38] using data from the baseline and posttest (8 weeks later). According to Shrout [39], there is no consensus on what is a good ICC. However, an ICC value of .60 or greater is considered acceptable [40, 41]. Convergent validity was evaluated using Spearman correlations (rho) to examine the relationships between the CERQ-k (total score and subscale scores) and measures of anxiety (trait anxiety subscale of STAI-C) and depression (CDI). These measures were selected based on previous studies [7, 8, 18, 42, 43]. The level of significance considered was .05. These statistical analyses were performed using SPSS v24.

## Results

### Confirmatory factorial analysis

CFA for the Spanish sample was carried out (*N* = 582) and yielded a nine-factor model coinciding with the original version of the CERQ-k [6]. The adjustment for the model was adequate: CFI = .94, TLI = .93, RMSEA = .05 (.052, .058). According to the criteria of Hu and Bentler [32], the CFA showed suitable fit for the 36-item measure because the indexes were greater than .90. The factors of the Spanish version of the CERQ are the same as the nine proposed by the original authors. Each factor is composed of 4 items, namely Self-Blame, Acceptance, Rumination, Positive Refocusing, Planning, Positive Reappraisal, Putting into Perspective, Catastrophizing and Other-Blame. All the items showed factor loadings greater or equal to .50, with the exception of items 18, 19 and 31(factor loadings were .49, .47 and .46, respectively) (Table 1).

**Table 1:** Confirmatory factor analysis: factor loadings (*N* = 582).

CERQ subscales	Factor loadings
**F1. Self-blame**	
I think that I am to blame	.80
I think that I have been stupid	.74
I think that it’s my own fault	.47
I think that it’s all caused by me	.66
**F2. Acceptance**	
I think that I have to accept it	.66
It just happened; there is nothing I can do about it	.55
I think that I can’t change it	.94
I think that I can’t do anything about it	.60
**F3. Rumination**	
Again and again, I think of how I feel about it	.65
I often think of what I am thinking and feeling about it	.54
All the time, I think that I want to understand why I feel that way	.63
I often think of how I feel about what happened	.58
**F4. Positive refocusing**	
I think of nicer things	.58
I think of nicer things that have nothing to do with it	.62
I think of something nice and not about what happened	.58
I think of nice things that have happened to me	.46
**F5. Planning**	
I think about what would be the best for me to do	.66
I think of how I can cope with it	.67
I think of how I can change it	.59
I think of what I can do best	.58
**F6. Positive reappraisal**	
I think that I can learn from it	.68
I think that it makes me feel ‘older and wiser’	.75
I think that there are good sides to it as well	.65
I think that it’s not all bad	.56
**F7. Putting into perspective**	
I think that worse things can happen	.85
I think that worse things happen to others	.63
I think that it’s not as bad as other things that could happen	.50
I think that there are worse things in the world	.54
**F8. Catastrophizing**	
I often think that it’s much worse than what happens to others	.78
Again and again, I think about how terrible it all is	.51
All the time, I think that this is the worst thing that can happen to you	.62
I often think about how horrible the situation was	.58
**F9. Other-blame**	
I think that others are to blame	.70
I think that others have been stupid	.49
I think that it’s the fault of others	.63
I think that it’s all caused by others	.64

### Analysis of items

Descriptive statistics of the items of the Spanish version of the CERQ-k were calculated. The corrected item-total correlations ranged from .22 to .68, which indicates a general adequate performance of the items. All item-correlations were above .30, except for item 20 (“I think that I can’t change it”). Since the removal of any item did not increase the ordinal alpha significantly, all items were kept. Table 2 shows the ordinal alphas, means, standard deviations and ranges for each questionnaire in the current study.

**Table 2:** Scale properties of the CERQ, CDI and Trait Anxiety Inventory (STAI-C).

	*α*	*M*	*SD*	range
Depression (CDI)	.82	9.40	6.25	0–39
Trait Anxiety Inventory (STAI-C)	.85	33.89	7.28	20–54
CERQ	.88	101.65	21.40	38–168
*Subscales*				
Self-blame	.65	9.92	3.50	4–20
Acceptance	.56	12.03	3.65	4–20
Rumination	.71	11.76	4.14	4–20
Positive refocusing	.75	12.62	4.27	4–20
Planning	.70	13.17	3.95	4–20
Positive reappraisal	.65	12.19	3.93	4–20
Putting into perspective	.67	11.99	4.03	4–20
Catastrophizing	.69	9.78	3.92	4–20
Other-blame	.70	8.16	3.38	4–20

*M* = Mean; *SD =* Standard Deviation; α = ordinal alpha.

### Internal consistency and test-retest reliability

The CERQ-k demonstrated acceptable internal consistency with coefficients ranging from .56 (Acceptance) to .75 (Positive Refocusing) for the nine scales (Table 2). Spearman correlations among the nine factors were calculated, and low values ranging from -.01 (Other-Blame and Positive Reappraisal) to .54 (Planning and Positive Reappraisal) were found. Most of these correlations were significant at *p* < .01, as shown in Table 3.

**Table 3:** Spearman correlations among CERQ scales and depression and anxiety problem scales.

	1	2	3	4	5	6	7	8	9
Self-blame	1								
2. Acceptance	.45**	1							
3. Rumination	.43**	.39**	1						
4. Positive refocusing	.18**	.26**	.29 **	1					
5. Planning	.40**	.40**	.42**	.48**	1				
6. Positive reappraisal	.31**	.38**	.35**	.47**	.54**	1			
7. Putting into perspective	.31**	.33**	.32**	.28**	.35**	.38**	1		
8. Catastrophizing	.37**	.24**	.43**	.12**	.18**	.11**	.28**	1	
9. Other-blame	.10*	.06	.19**	.06	.06	-.01	.14**	.44**	1
Depression (CDI)	.19**	-.01	.26**	-.18**	-.16**	-.09	.08	.30**	.16**
Trait Anxiety Inventory (STAI-C)	.25**	.06	.30**	.004	.004	.03	.11	.27**	.16**

**Correlation is significant at the .01 level (2-tailed).

*Correlation is significant at the .05 level (2-tailed).

A subsample of 211 children (36.25% of the sample) was asked to complete the CERQ-k 8 weeks later in order to examine test-retest reliability, according to Domínguez-Sánchez et al. [14] in the Spanish validation of the CERQ for adults. ICC test-retest coefficients were low to moderate with the following values: .65 for Self-Blame, .54 for Acceptance, .70 for Rumination, .67 for Positive Refocusing, .60 for Planning, .65 for Positive Reappraisal, .63 for Putting into Perspective, .63 for Catastrophizing, and .56 for Other-Blame. These ICC coefficients indicated that the test-retest reliability was acceptable, except for the Acceptance subscale, whose value did not reach the recommended cut off [44]. The test-retest coefficient for the total scale score was adequate (ICC = .74).

### Convergent validity

To evaluate convergent validity of the CERQ-k, a subsample of 278 children (47.76% of the sample) was used. Spearman correlation analyses showed significant negative relationships between depression and the adaptive cognitive emotion regulation strategies of positive refocusing and planning. By contrast, evidence of convergent validity was found through significant positive correlations among the CDI and maladaptive CER strategies (Self-Blame, Rumination, Catastrophizing, and Other-Blame subscales of the CERQ-k), and among the STAI-C trait anxiety subscale and the Self-Blame, Rumination, Catastrophizing, and Other-Blame subscales of the CERQ-k. These results indicate that greater use of the cognitive emotion regulation strategies of self-blame, rumination, catastrophizing, and other-blame were associated with higher levels of depression and anxiety symptomatology. However, using the cognitive emotion regulation strategies of positive refocusing and planning was associated with lower levels of depression.

## Discussion

The current study tested the psychometric properties and factor structure of the Spanish version of the CERQ-k in a community sample of children aged from 7 to 12 years old. The CFA demonstrated an excellent fit and confirmed the original nine-factor structure of the CERQ-k [6] and the adapted Chinese version for children [8]. The CER strategies are self-blame, acceptance, rumination, positive refocusing, planning, positive reappraisal, putting into perspective, catastrophizing, and other-blame. Thus, the Spanish version of the CERQ-k maintains the same nine scales as the original 36-item version [2, 6] that has also been validated with adult populations across several countries: France [20], China [21], Romania [22], Iran [23], Turkey [24], Spain [14], Argentina [25] and Peru [26], among others. According to Field [45], when item-correlation coefficient is above .30 is expected a positive correlation between that specific item and the factor. However, a low item-correlation indicates that the item does not measure the same construct than other items included in the same factor. Given the item-correlation coefficients for the CERQ-k were above .30 (except for item 20) and factor loadings had medium-to-high magnitude, all items were retained.

The internal consistency reliability of the subscales was generally good, with alphas ranging from .56 to .75. These results are similar to other validation studies that have used the CERQ-k in a sample of 9-11-year-old 717 primary school youngsters in the Netherlands (from .62 to .79) [6, 7]. When the scale presents multidimensional nature, such as CERQ-k that includes four items per factor, it is reasonable that internal consistency is moderate-adequate [45]. Test-retest reliability was also examined over an interval of 8 weeks, and data established a satisfactory total scale test-retest reliability index (ICC = .74) and moderate to acceptable correlations for the subscales (ICC = .54 to .70), which suggests that the CER strategies can be considered relatively stable styles, although less than others such as personality traits [6]. Consistently, Liu et al. [8] found test-retest coefficients ranging from .53 to .70 over one month. This finding also suggests that CER strategies may vary depending on the situations children face and the context in which the unpleasant event unfolds [13].

In addition, the construct validity of the Spanish version of the CERQ-k was verified. Consistent with empirical studies using CERQ-k in children [14, 15, 25] and adults [17, 26] and a meta-analytic review on emotion-regulation strategies across psychopathology [15], the subscales corresponding to less adaptive CER strategies (Rumination, Self-Blame, Catastrophizing, and Other-blame) were more related to depression and anxiety symptoms, supporting the convergent validity of the CERQ-k. The results suggest that children who use maladaptive CER strategies (including Rumination, Self-Blame, Catastrophizing, and Other-blame) are more likely to develop depressive and anxiety symptomatology. Convergent validity was also demonstrated by the relationships between the adaptive CER strategies (positive refocusing and planning) and presence of fewer symptoms of depression. Therefore, positive refocusing and planning would act as strategies that have a positive effect on the prevention of depression in children. The reason why no further stronger negative correlations were found between adaptive strategies, anxiety and depression remains unclear. However, our findings are consistent with existing studies conducted with Chinese [8], Dutch [6, 13], and Spanish-Speaking children and youth [18], which found the strongest negative associations with anxiety and/or depression only with positive refocusing and/or positive reappraisal. Only the study with a Chinese sample [8] has found significant negative associations between most adaptive CER strategies and depression, including positive refocusing and planning as found in our study. In this regard, it is necessary further research with children from different settings to determine possible cultural differences in the concept of cognitive emotional regulator, the use of CER strategies, and their impact on emotional problems since cognitive coping styles may be more or less adaptive depending on the circumstances in which they are assessed [13]. In adults, the research is more extensive and the cross-cultural stability between the presence of these emotional problems and the use of CER strategies has been highlighted, and only minor differences have been found [14]. For instance, as Domínguez-Sánchez et al. [14] noted, compared to individualistic societies (e.g., Spain), in collectivistic societies (e.g., China) there has been found a greater relationship between anxiety and blaming others or a greater use of strategies such as positive refocusing instead of positive reappraisal. Lastly, the results found in this study suggest that more research is warranted with Spanish children from different areas of Spain and using different measures of childhood anxiety and mood problems to determine the relationships between the CER strategies and these emotional problems, as well as to confirm the psychometric properties of the adapted scale.

### Limitations

The study has some limitations that have to be considered. The research used self-report measures to collect the data, so it would be advisable to collect information also from parents to compare scores between both measures. Additionally, the lack of studies on the psychometric properties of different versions of the CERQ-k made it difficult to compare the results of this study with data collected in other countries. Despite these limitations, findings of this study confirm that the Spanish version of the CERQ-k constitutes a reliable and valid instrument for measuring strategies of cognitive emotion regulation in Spanish children.

### Practical implications

Although some studies have examined the suitability of the CERQ for use with children (CERQ-k), the present study is the first that has adapted the CERQ for use with children from Spanish-speaking countries. Therefore, this research provides an easier tool to administer in childhood in order to explore CER strategies. This instrument can be useful for researchers and clinicians to know the existence of maladaptive CER patterns that may increase the risk of emotional problems and orient interventions and prevention of mental health problems in children. Future research should focus on a shorter version of the CERQ-k, and the validation of this tool for Spanish adolescents in order to provide researchers and clinicians with a tool to evaluate CER in this population.

To summarize, the results of the current study, together with previous international studies [6, 8], provide preliminary evidence for the validity and reliability of the CERQ for children as a multidimensional tool of CER. This study offers a valuable instrument to early detection of emotional disorders and to guide the psychological treatments in Spanish-speaking children that it can be used in research large-scale studies and at clinical settings.
